# New Insights into Bitopic Orthosteric/Allosteric Ligands of Cannabinoid Receptor Type 2

**DOI:** 10.3390/ijms24032135

**Published:** 2023-01-21

**Authors:** Rebecca Ferrisi, Beatrice Polini, Caterina Ricardi, Francesca Gado, Kawthar A. Mohamed, Giovanna Baron, Salvatore Faiella, Giulio Poli, Simona Rapposelli, Giuseppe Saccomanni, Giancarlo Aldini, Grazia Chiellini, Robert B. Laprairie, Clementina Manera, Gabriella Ortore

**Affiliations:** 1 Department of Pharmacy, University of Pisa, 56126 Pisa, Italy; 2 Department of Pathology, University of Pisa, 56126 Pisa, Italy; 3 Department of Pharmaceutical Sciences, University of Milan, 20133 Milano, Italy; 4 College of Pharmacy and Nutrition, University of Saskatchewan, Saskatoon, SK S7N 5E5, Canada; 5 Department of Pharmacology, College of Medicine, Dalhousie University, Halifax, NS B3H 4R2, Canada

**Keywords:** drug discovery, docking, endocannabinoid system, cannabinoid receptor type 2, allosteric modulators, dualsteric/bitopic, anti-inflammatory activity, human microglial cells

## Abstract

Very recently, we have developed a new generation of ligands targeting the cannabinoid receptor type 2 (CB2R), namely **JR** compounds, which combine the pharmacophoric portion of the CB2R positive allosteric modulator (PAM), **EC21a**, with that of the CB2R selective orthosteric agonist **LV62**, both synthesized in our laboratories. The functional examination enabled us to identify **JR14a**, **JR22a**, and **JR64a** as the most promising compounds of the series. In the current study, we focused on the assessment of the bitopic (dualsteric) nature of these three compounds. Experiments in cAMP assays highlighted that only **JR22a** behaves as a CB2R bitopic (dualsteric) ligand. In parallel, computational studies helped us to clarify the binding mode of these three compounds at CB2R, confirming the bitopic (dualsteric) nature of **JR22a**. Finally, the potential of **JR22a** to prevent neuroinflammation was investigated on a human microglial cell inflammatory model.

## 1. Introduction

G protein-coupled receptors (GPCRs) are the most intensively studied drug targets, and the GPCR drug discovery landscape continues to offer enormous opportunities for new and much-improved drugs. The long track record of their success has been largely dominated by compounds targeting the same site as the endogenous ligand (i.e., orthosteric binding site), thus narrowing GPCR-ligand pharmacology to the concepts of agonism and antagonism. The need to address issues of target and pathway selectivity of GPCRs, of which orthosteric ligands are often deficient due to highly homologous receptor orthosteric sites, has encouraged the search for new paradigms for GPCRs selectivity: the targeting of allosteric sites [1] and the exploitation of biased agonism [2]. The dynamic picture of GPCR regulation increasingly involves the discovery of allosteric modulators, which bind to topographically distinct and less evolutionarily conserved binding sites than the orthosteric ones, modifying the binding and/or signaling of orthosteric ligands. Allosteric mechanisms can be targeted to improve safety in certain biological circumstances characterized by off-target effects, poor selectivity, and the abrogation of temporal and/or spatial aspects of endogenous physiological signaling. “Functionally selective” or “biased” agonists can preferentially engage one signaling pathway over others, and this may translate into different physiological responses aimed at improving therapeutic outcomes and, in the meanwhile, avoiding unwanted side effects [3]. Biased agonism is an integral part of the allosteric nature of GPCRs, as allosteric ligands favor unique receptor conformations and signaling patterns resulting from orthosteric ligands.

Besides these two trends in GPCR pharmacology, in the past few years, bitopic ligands have emerged as a “new” frontier to obtain selective targeting of GPCRs by covalently connecting orthosteric and allosteric pharmacophores. Bitopic ligands are an extension of the bivalent ligand approach, according to which they exhibit two distinct pharmacophores joined by a linker in a single chemical entity [4,5]. In this specific case, a bitopic ligand would ideally be capable of simultaneously interacting with orthosteric and allosteric binding sites on the same receptor. Theoretically, several reasons justify the choice of a bitopic ligand: (i) the greater affinity for the target GPCR due to the binding to the orthosteric site; (ii) the improved tissue and receptor selectivity profile, owing to the targeting of an allosteric site; (iii) the promotion of stimulus bias; (iv) unlike allosteric modulators, they do not need a proper endogenous agonist tone which could be reduced (e.g., in neurodegenerative disorders); (v) the possibility to achieve all these advantages through the use of a single biologically active molecule [6].

On the other hand, the design of bitopic ligands raises important challenges, such as the choice of the linker moiety, the unequivocal demonstration of bitopic pharmacology, as well as some “druggability” issues due to the large size and molecular weight that may negatively impact bioavailability and, thus, in vivo utility.

**Figure 1:**
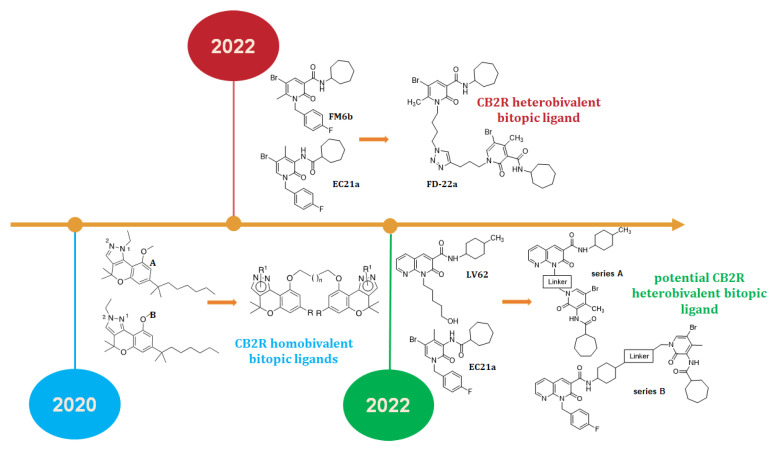
Timeline of published studies about CB2R bitopic (dualsteric) ligands.

Many examples of engineered bitopic ligands have been described for GPCRs, including the muscarinic receptors [7] and the adenosine receptors [8]. However, so far, only two examples have been reported in the literature for the cannabinoid receptor type 2 (CB2R): in particular, the first one was reported by Morales and colleagues, who synthesized the first CB2R homobivalent bitopic ligands, linking the chromenopyrazole derivatives **A** and **B** (Figure 1), differing in the position of the N-ethyl at the pyrazole ring. Although the pharmacophoric units of these homobivalent ligands are derived from CB2R orthosteric agonists, it has been demonstrated that the proposed symmetrical bivalent compounds are recognized by both the orthosteric site and the vestibule/exosite of CB2R, thus highlighting a bitopic binding mode [9].

More recently, our research group has described the first CB2R heterobivalent bitopic ligand, **FD22a** (Figure 1) [10], which combines the pharmacophoric portions of the CB2R positive allosteric modulator, **EC21a** (Figure 1) [11], and the CB1R/CB2R orthosteric agonist **FM6b** (Figure 1) [12,13], through an alkyl chain characterized by the presence of a 1,2,3-triazole ring. **FD22a** meets requirements typical of a bitopic ligand, such as receptor-subtype selectivity and biased signaling for cAMP inhibition versus βarrestin2 recruitment. In addition, to give further validation that the observed pharmacology was coherent with the expectations of a bitopic agonist model, **FD22a** was exposed to co-administration experiments in the presence of the CB2R PAM **EC21a** or the CB2R antagonist/inverse agonist SR144528. In parallel, docking studies allowed us to validate a computational model for **FD22a**, highlighting the concomitant association with both orthosteric and allosteric sites on CB2R [10].

Even more recently, we have presented two sets of novel potential CB2R heterobivalent bitopic ligands, series **A** and **B** (Figure 1), connecting the pharmacophoric portion of the CB2R PAM **EC21a**, with that of the CB2R selective orthosteric agonist **LV62** (Figure 1), previously synthesized by us [14]. The two series of novel compounds differ in the site of attachment of the linker at the level of the **LV62** moiety, namely the nitrogen atom at position 1 for series **A** and the 4-methyl cyclohexyl group at position 3 for series **B** (Figure 1) [15]. 

**Figure 2:**
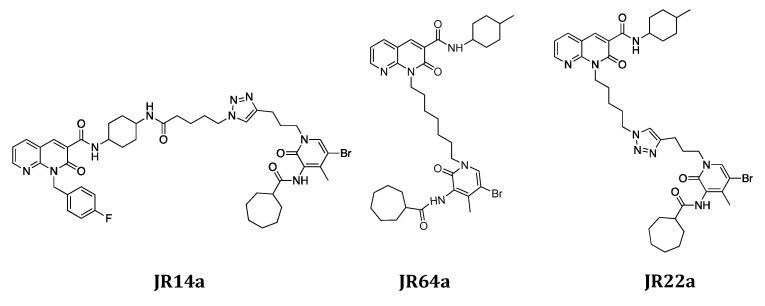
Structures of **JR14a**, **JR64a**, and **JR22a**.

The complete panel of new orthosteric/allosteric hybrid CB2R ligands was evaluated to measure their ability to inhibit cAMP accumulation and/or to enhance βarrestin2 recruitment, highlighting for most of the novel compounds a significant signaling ‘bias’ in favor of G protein activation over βarrestin2 recruitment [15]. In particular, **JR64a** and **JR22a** derivatives have been identified as the most promising compounds of series **A** while **JR14a** resulted in the best compound of series **B** (Figure 2) [15]. Notably, **JR22a** is structurally similar to the previously identified CB2R heterobivalent bitopic ligand **FD22a** since both compounds present the same linker connecting the two pharmacophoric groups [10]. 

In the current study, to further expand our knowledge on the bitopic nature of newly designed CB2R ligands, additional cAMP assays were carried out. Collected data highlighted that exclusively **JR22a** behaves as a CB2R bitopic (dualsteric) ligand. In addition, computational studies significantly contributed to confirming a bitopic binding mode for **JR22a** inside CB2R. Finally, the anti-inflammatory action of **JR22a** was investigated by using LPS/TNFα stimulated human microglial clone 3 cell line (HMC3).

## 2. Results and Discussion

### 2.1. Inhibition of Forskolin-Stimulated cAMP

#### 2.1.1. Evaluation of the Allosteric Interaction

**Figure 3:**
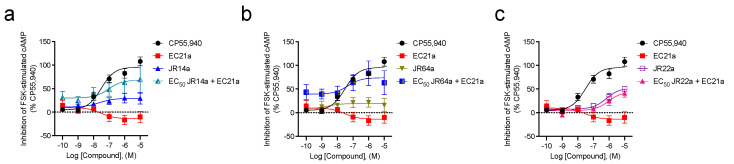
CB2R-dependent inhibition of FSK-stimulated cAMP in CHO cells stably expressing *h*CB2R. cAMP inhibition data are expressed as the %CP55,940 response. Cells were treated with ligands simultaneously, as indicated. 53 nM **JR14a** (**a**), 75 nM **JR64a** (**b**), and 100 nM **JR22a** (**c**) were chosen after the completion of preliminary experiments with compounds alone for ease of calculations to approximate the EC_50_ for each compound alone. Data were fitted to a nonlinear regression (three-parameter model, GraphPad v. 9.0). Data are mean ± S.E.M. 3 or more independent experiments performed in triplicate. Specific *n* values and data from these graphs are presented in Table 1.

To directly probe the ability of **JR14a**, **JR64a**, and **JR22a** to interact with the CB2R allosteric site, the effect of the combination of EC_50_ **JR14a**, **JR64a**, or **JR22a** with 0.1 nM–10 μM **EC21a** on the inhibition of FSK-stimulated cAMP accumulation was investigated (Figure 3, Table 1). For these additional experiments, modest differences in potency and efficacy were noted between these data (Table 1) and previously reported results [15]; however, these differences were all within experimental and technical variability and, therefore, not significantly different for CP55,940, **JR14a, JR64a**, or **JR22a**. For **EC21a,** we previously did not observe a concentration response within the concentration range used [15]; in contrast, the present data showed a small downward curve in the **EC21a** response (pEC_50_ = 7.5) (Table 1). Importantly, the previous and current E_max_ data for **EC21a** are not different from 0 or each other, affirming the conclusion that **EC21a** alone was inactive (Table 1) [15]. In the case of **JR14a** (Figure 3a, Table 1), the data demonstrated that the baseline and E_max_ both increase in the combination treatment (E_max(JR14a)_ = 29 ± 5; E_max(JR14a + EC21a)_ = 68 ± 10), while the EC_50_ is not significantly different from **JR14a** alone (pEC50_(JR14a)_ = 7.6; pEC_50(JR14a + EC21a)_ = 7.0). The results are congruent with a positive allosteric behavior of **EC21a** for **JR14a** since **EC21a** augments the efficacy (but not the potency) of **JR14a**. We can make the same remarks for **JR64a** (Figure 3b, Table 1). While the potency of **JR64a** is not increased by the PAM **EC21a** (pEC_50(JR64a)_ = 8.6; pEC_50(JR64a + EC21a)_ = 7.3), we can detect a significant shift upward in the EC_50_ **JR64a** + **EC21a** curve, in line with an increase in the maximum response compared to that produced by **JR64a** alone (E_max(JR64a)_ = 19 ± 6; E_max(JR64a + EC21a)_ = 74 ± 10). Therefore, there are compelling data also for a PAM effect of **EC21a** against **JR64a**. In both cases, the obtained data might suggest that either **JR14a** and **JR64a** are primarily acting through the orthosteric site or that **JR14a**, **JR64a,** and **EC21a** have different allosteric sites. A different evaluation applies instead to **JR22a**. As shown in Figure 3c, the results indicate that the activity of **JR22a** doesn’t seem to be augmented in the presence of the PAM **EC21a**, both in terms of potency (pEC_50(JR22a)_ = 6.2; pEC_50(JR22a + EC21a)_ = 6.0), and efficacy (E_max(JR22a)_ = 53 ± 6; E_max(JR22a + EC21a)_ = 44 ± 9) (Table 1). This could indicate that **JR22a** and **EC21a** share the same allosteric site suggesting a bitopic orthosteric/allosteric interaction of **JR22a**.

#### 2.1.2. Evaluation of the Orthosteric Interaction

**Figure 4:**
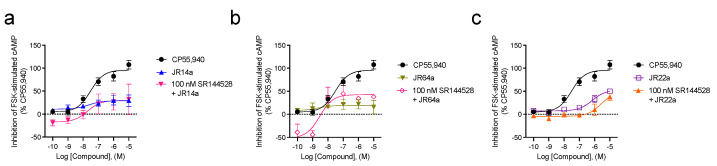
CB2R-dependent inhibition of FSK–stimulated cAMP CHO cells stably expressing *h*CB2R. cAMP inhibition data are expressed as the %CP55,940 response. Cells were treated with ligands simultaneously, as indicated. Addition of 100 nM SR144528 to **JR14a** (**a**), **JR64a** (**b**), or **JR22a** (**c**). Data were fitted to a nonlinear regression (three-parameter models, GraphPad v. 9.0). Data are mean ± S.E.M. 3 or more independent experiments performed in triplicate. Specific *n* values and data from these graphs are presented in Table 1.

We next investigated the effect of the combination of 0.1 nM−10 μM **JR14a**, **JR64a**, and **JR22a** compounds against 100 nM CB2R antagonist/inverse agonist **SR144528** (Figure 4, Table 1). As the concentration of **JR14a** (Figure 4a), **JR64a** (Figure 4b), or **JR22a** (Figure 4c) is increased, the effects of **SR144528** are effectively reversed. This latter finding suggests that all three compounds can adopt a binding mode on the receptor that certainly involves attachment to the orthosteric site. Importantly, the concentration-dependent inhibition of cAMP supports the activity of these compounds at *h*CB2R.

Overall, these data indicate that both **JR14a** and **JR64a** are predominantly acting at the orthosteric site as agonists whose activities are augmented by the PAM **EC-21a** and inhibited by **SR144528**. Further, the results support that **JR22a** interacts with both orthosteric and allosteric sites (i.e., bitopic), thus reflecting the same bitopic mode of orthosteric/allosteric interaction previously established for the CB2R heterobivalent bitopic ligand **FD22a** [10].

### 2.2. Anti-Inflammatory Activity of Selected CB2R Heterobivalent Bitopic Ligand (JR22a) on Human Microglial Clone 3 Cell Line (HMC3)

Given the functional evidence for a bitopic interaction with CB2R demonstrated by the analog **JR22a**, we subsequently wanted to evaluate its anti-inflammatory properties.

**Figure 5:**
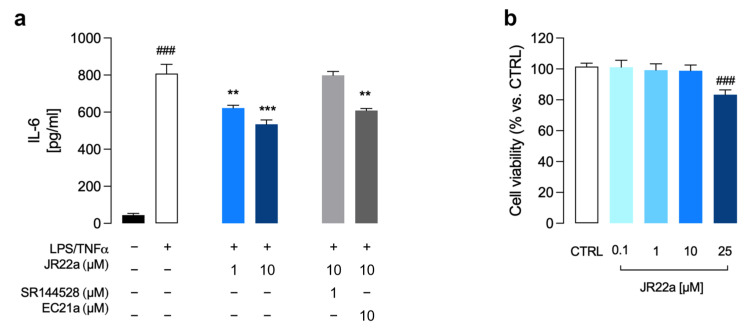
Effects of **JR22a** on the inflammatory response (**a**) and cell viability (**b**) in HMC3 cells. (**a**) Release of inflammatory IL-6 after exposure of HMC3 cells to LPS (10 μg/mL)/TNFα (50ng/mL) stimulus for 24 h. Data represent means ± S.E.M. from *n* = 3 independent experiments performed in duplicate. Statistical analysis was performed by ordinary one-way ANOVA followed by Tukey’s multiple comparison test. ^###^ *p* < 0.005 compared to control cells; ** *p* < 0.01, *** *p* < 0.005 compared to LPS/TNFα stimulated HMC3 cells. (**b**) MTT assay was performed with different concentrations of **JR22a**. Data represent means ± S.E.M. from *n* = 3 independent experiments performed in triplicate. Statistical analysis was performed by ordinary one-way ANOVA followed by Tukey’s multiple comparison test. ^###^ *p* < 0.005 vs. control.

The anti-inflammatory response of analog **JR22a** was examined using LPS/TNFα stimulated HMC3 cells, a model of inflammation previously used by us to evaluate the anti-inflammatory properties of **JR14a** and **JR64a** derivatives [15]. Consistent with our previous findings [10,15], LPS/TNFα administration induced a significant release of pro-inflammatory interleukin 6 (IL-6) in cell media compared to control cells (Figure 5A).

In order to evaluate the ability of **JR22a** to prevent the inflammatory response, HMC3 cells were pre-treated with selected concentrations (1 and 10 μM) of test compound for 30 min, followed by LPS/TNFα exposition. After 24 h, the release of inflammatory marker IL-6 was measured by using an ELISA assay. As shown in Figure 5A, **JR22a** induced a significant dose-dependent decrease in IL-6 levels in the cultured medium. Noteworthy, pre-administration with CB2R selective antagonist **SR144528** (1 μM) almost completely abolished the **JR22a** effect on IL-6 release (Figure 5A), confirming a truly CB2R-mediated anti-inflammatory activity of test compound **JR22a**.

To further clarify for **JR22a,** a CB2R bitopic character, we performed co-administration experiments using the positive CB2R allosteric modulator **EC21a** at equimolar doses (10 µM). As shown in Figure 5A, co-treatment with **EC21a** did not interfere with the ability of **JR22a** to prevent IL-6 release after LPS/TNFα stimulus in HMC3 cells, supporting a bitopic role of test compound **JR22a**.

Finally, no cytotoxic effect was detected in HMC3 cells after treatment with **JR22a** at selected concentrations (1 and 10 μM) (Figure 5B), whereas when used at 25 μM, significant inhibition of cell viability was observed (Figure 5B). 

### 2.3. Stability of JR22a

#### 2.3.1. Plasma Stability

**Figure 6:**
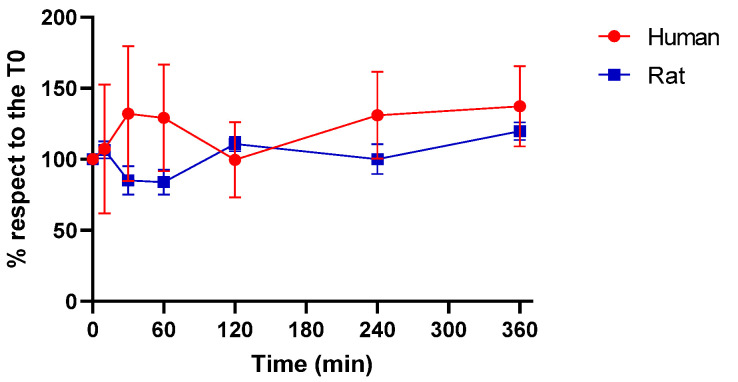
Rat and human plasma stability of **JR22**: the compound was stable up to 360 min. Results are reported as mean relative % ± SD (*n* =3).

Plasma stability was evaluated at the time points 0, 10, 30, 60, 120, 240, and 360 min over two species, rodent (rat) and human, and assessed by LC-HRMS. Results are expressed as a relative percentage (mean ± SD) with respect to the amount at t = 0 min. **JR22a** is stable both in rat and human plasma up to the last time point tested (360 min), as can be observed in Figure 6.

#### 2.3.2. Computational Metabolism Prediction

**JR22a** metabolism was predicted using MetaSite software [16]. Potential hepatic metabolites were estimated using a list of 40 common cytochromes P450 biotransformations, including hydroxylation, dealkylation, carbonylation, and dehalogenation. For each potential site of metabolism, the software assigned a probability score. The most probable second- and third-generation metabolites were predicted for the top five first-generation metabolites, with the aim of evaluating the possibility of a bond cleavage of the **JR22a** scaffold. MetaSite predicted only one site of metabolism scoring 100% (see Figure S2), corresponding with the methyl in position 4 of the oxopyridine moiety, where aliphatic hydroxylation, oxidation, or dehydrogenation can occur. Within the further-generation metabolites, only one product of cleavage was detected at the third generation, at the C-N bond of the 4-methylcyclohexylcarboxamide moiety (Figure S2). These results confirm the hepatic stability of the bitopic structure with respect to the metabolic fragmentation in two distinct orthosteric and allosteric parts.

### 2.4. Computational Studies

Starting from our previous definition of the potential allosteric binding site of CB2R and from docking results obtained for the compounds of the **FD** series [10], we conducted a computational study with the aim of validating the binding pose of our bitopic compounds in CB2R and adding new details on the structure-activity relationship for the bitopic behavior of our CB2R ligands. We applied the procedure already described for the 2-oxo-pyridine derivatives of the series **FD** [10] to the **JR64a**, **JR14a**, the most interesting 1,8-naphthyridin-2-one derivatives of series **A** and **B**, respectively, and to **JR22a** of the series **A** for a comparison with the structural analog ligand **FD22a** previously described by us as a CB2R heterobivalent bitopic ligand [10]. In fact, both these last compounds share the same linker between pharmacophoric orthosteric and allosteric portions. We first performed the docking of **JR** compounds in the cryo-EM structure of CB2R-Gi protein in complex with agonist WIN 55,212-2 (PDB ID: 6PT0) [17]. Then, to overcome the scaffold constraint used during the docking calculation and allow all compounds to reach a stable free conformation, a molecular dynamics (MD)simulation was performed. All complexes show good stability in terms of root-mean-square deviation (RMSD) of α-carbons and ligands heavy atoms (Figure S3). Some fluctuations in the backbone steadiness were registered, even if without exceeding 2 Å of RMSD, in association with a Trp258 [18] conformation variability in **JR22a** and **JR14a** complexes. All simulations were compared, analyzing the ligand disposition to the main interactions with the binding site and the largest deviation in residue conformation during dynamics. At the same time, for a comparison between the disposition of the allosteric portion of the bitopic ligands and that of the PAM **EC21a** in CB2R, we applied the same computational procedure to the complex ternary CP55,940-**EC21a**-CB2R. In this way, we aimed to highlight the key interactions and the role of **EC21a** in CB2R. CP55,940 was used as an orthosteric ligand since **EC21a** was previously shown to increase the binding of [^3^H]CP55,940 to CB2R and to enhance the ability of CP55,940 to stimulate [^35^S]GTPγS binding to CB2R, thus demonstrating to be a CB2R PAM [11].

**Figure 7:**
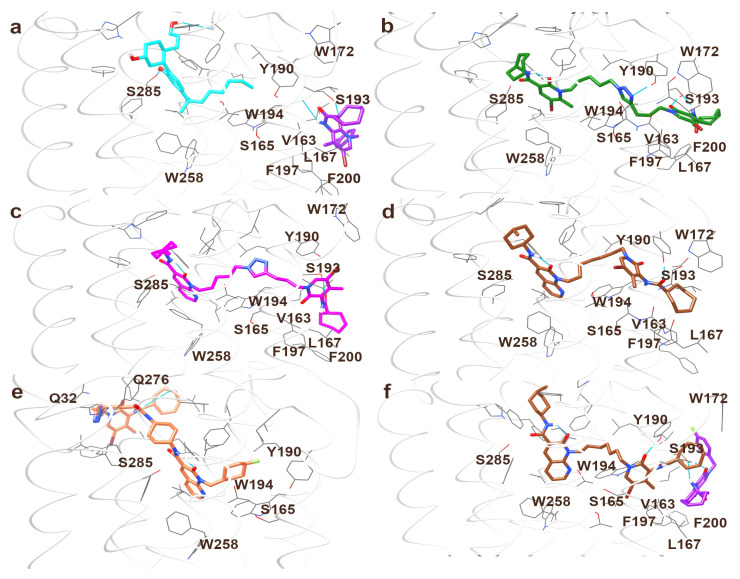
Optimized docking poses of: (**a**) CP55,940 and **EC21a** in ternary complex with CB2R; (**b**) **FD22a**, (**c**) **JR22a**, (**d**) **JR64a**, (**e**) **JR14a** in complex with CB2R; (**f**) **JR64a** and **EC21a** in ternary complex with CB2R. Hydrogen bonds are represented in cyan lines. The ribbon of residues 162–172, near the putative allosteric site, is removed in (**a**–**d**) for clarity.

All results are reported in Figure 7 and Figure S3. **EC21a** interacts with the surface of TM5 (Figure 7), in particular, engages a strong hydrogen bond with Ser193, which is determining for its permanence in this site; two aromatic interactions involve the oxopyridine and fluorophenyl moieties of **EC21a** with Phe197 and Phe200, respectively; a lipophilic stabilization of cycloptyl-ring is due to Leu163, Val164 and Leu167 during the dynamics simulation. Interestingly, this ligand disposition allows a 4.9 Å averaged distance between the amidic carbonyl of **EC21a** and the hydroxyl group of Tyr190, with the engagement of a hydrogen bond in some frames. Residue Tyr190 is inside the receptor, and it is part of the orthosteric binding site. Phe197 and Val164, in spite of their location on the surface of TM5, are involved in aromatic and lipophilic networks with Trp194, which interacts directly with the orthosteric agonist, and Ser165, which moves during the simulation losing the hydrogen bond with TM3. The aim of our MD simulation was docking validation; further simulations are needed to understand the remote connection between **EC21a** docking and allosteric modulation. This preliminary study shows that the presence of **EC21a** at an allosteric binding site on the surface of TM5 may produce effects on residues within the core of CB2R.

**JR22a** binds CB2R in a similar mode as its analog **FD22a** (for comparison, Figure 7c,b, respectively). In the orthosteric portion, the intramolecular hydrogen bond involving the exocyclic amine stably guarantees the amide planarity and favors the polar interaction with Ser 285, especially in the **FD22a** complex. The aliphatic ring is quite flexible, in particular in **FD22a**, which in fact, shows a higher RMSD of the ligand-heavy atoms during the simulation (see Figure S3). The naphthyridine ring of **JR22a** is rotated and shifted to about 2Å with respect to a 2-oxopyridine moiety of **FD22a** (orthosteric portion) (see Figure S4). This position allows a good aromatic stacking with Phe117 and prevents the approach of Trp258, but it is not well superposed with the orthosteric moiety of the crystallographic ligand [17] and weakens the interaction with Ser285. This assessment produces a slight instability during the MD simulation of Trp258 conformation, which was not switched but produced fluctuant deformations in the helices conformations, leading to a higher RMSD of alpha-carbons (anyway lesser of 2Å) with respect to the **JR22a** complex (Figure S3). This is an agreement with the lower affinity of **JR22a** for CB2R, showing a Ki of 95 nM (see Figure S1) vs. Ki of 1, 4 nM for **FD22a** [10]. As a consequence of the slightly different orthosteric pose, the linker assumes a divergent direction and promotes, within the variability of the allosteric portion on the surface of the receptor, a different inclination of the 2-oxopyridine moieties of **FD22a** and **JR22a**. In both cases, the interaction of the amide with Ser193 is preserved via NH as a hydrogen bond donor in **FD22a** (in addition to the interaction of Ser193 with the carbonyl of the 2-oxopyridine) or via carbonyl oxygen of **JR22a** as an acceptor.

A significant difference is related to Tyr190: in **FD22a,** this residue retains about the same conformation as in the crystallographic structure of CB2R [17] and engages a hydrogen bond with the triazole ring, while in **JR22a,** it rotates towards the outer of TMs. Also, the aromatic environment differs between **FD22a** and **JR22a** in the allosteric region: the 2-oxopyridine position moiety is surrounded by Trp172 and Phe197 in **FD22a**, while **JR22a** lies parallel to TM5 without significant interactions. 

Figure 7d presents the results of **JR64** docking: the orthosteric portion of **JR64a** presents the same orientation as **JR22a**, but the different and shorter linker cannot reach Ser193 for hydrogen bonding. Tyr190, without any rotation with respect to the crystallographic structure [17], engages a hydrogen bond with the distal amide instead of Ser193; the 2-oxopyridine moiety occupies about the same position as triazole in **JR22a**-CB2R complex, and the potential allosteric site remains empty. It’s different from the situation of **JR14a** (Figure 7e): the linker attachment on the N1 nitrogen of the naphthyridine nucleus completely changes the orientation of the ligand in CB2R. The binding of the orthosteric portion is preserved, but the linker is positioned across the opposite transmembrane region with respect to **JR22a** and **JR64a**, carrying the pseudo-allosteric portion on the receptor surface between TM1 and TM7, adjacent to the site of cholesterol CLR403 of the 6PT0 crystallographic structure [17] where interacts with Gln32 and Gln276. Some attempts to dock **JR14a** in a reversed binding mode, positioning the linker towards TM5, did not give plausible results. Very interesting, in this case, Ser165 preserves the hydrogen bond with TM3.

While the putative allosteric region appears empty in the **JR14a** binding pose, there could be a certain overlap of the cycloheptyl tail of **JR64a** with the **EC21a** site. To verify that in this condition, **EC21a** is still able to bind to the allosteric site, a further simulation was performed on the complex ternary **JR64a**-**EC21a**-CB2R, resulting in Figure 7f. In spite of the flexibility due to the ligand binding on the receptor surface, the pose of **EC21a** in the ternary complex with CP55,940 is quite similar to the one with **JR64a** and maintains the same interactions of the allosteric tails of **JR22a**.

These preliminary results are coherent with the ability of **JR22a**, similar to **FD22a** [10], to fill both orthosteric and putative allosteric sites, engaging the same key interactions of the single orthosteric and allosteric ligands. In contrast, the shorter **JR64a** linker cannot reach the allosteric site effectively and allows simultaneous interaction with **EC21a**. Instead, **JR14a**, in view of the linker attachment on the N1 nitrogen of naphthyridine, protrudes towards TM7 and TM1 in a region already described for binding homobivalent bitopic ligands [9]. Further studies are needed to understand the role of some residues highlighted in the course of our MD simulations in networking the allosteric modulation.

## 3. Materials and Methods

### 3.1. Reagents and Cell Lines

CP55,940 was purchased from Cayman Chemicals (Ann Arbor, MI, USA). [^3^H]CP55,940 (174.6 Ci/mmol) was purchased from PerkinElmer (Guelph, ON, Canada), whereas SR144528 was from Tocris (Bristol, UK). Chinese hamster ovary (CHO)-K1 cells untransfected or stably-expressing human cannabinoid CB2R (*h*CB2R) were used as previously reported [19,20]. Cells were maintained at 37 °C, 5% CO_2_ in F-12/DMEM containing 1 mM _L_-glutamine, 10% fetal bovine serum (FBS), and 1% Pen/Strep and hygromycin B (300 μg/mL) and G418 (400 μg/mL). HitHunter^®^ (cAMP) CHO-K1 cells stably expressing *h*CB2R from DiscoveRx (Eurofins, Fremont, CA, USA) were maintained at 37 °C, 5% CO_2_ in F-12 DMEM containing 10% FBS and 1% Pen/Strep with 800 μg/mL geneticin.

The human microglial clone 3 cell line (HMC3) (ATCC^®^ CRL-3304™) was cultured in high glucose DMEM supplemented with 10% FBS, streptomycin (100 g/mL) and penicillin (100 U/mL) (Sigma-Aldrich, Milan, Italy). LPS (Cat. number L4391) and TNFα (Cat. number H8916) were purchased from Sigma-Aldrich (Milan, Italy).

### 3.2. HitHunter cAMP Assay

This method was conducted as described previously [19,20]. The quantification of FSK-stimulated cAMP accumulation was performed using the DiscoveRx HitHunter assay. Twenty thousand cells/well were plated in low-volume 96-well plates and incubated overnight in Opti-MEM containing 1% FBS at 37 °C and 5% CO_2_. Opti-MEM media was then removed and replaced with cell assay buffer (DiscoveRx), and cells were co-treated at 37 °C with 10 μM FSK and ligands for 90 min. The cAMP antibody solution and cAMP working detection solutions were added to cells (DiscoveRx), and cells were incubated for 60 min at room temperature. cAMP solution A (DiscoveRx) was added, and cells were incubated for an additional 180 min at room temperature before chemiluminescence was measured on a Cytation5 plate reader (top read, gain 200, integration time 10,000 ms). 

### 3.3. Analysis of Interleukin 6 (IL-6) Release in HMC3 Cells 

The pro-inflammatory IL-6 levels were evaluated by a specific ELISA assay (RAB0306, Sigma-Aldrich, Milan, Italy) on collected culture media. Briefly, HMC3 cells were exposed to pretreatment with a test compound for 30 min followed by LPS (10 μg/mL)/TNFα (50 ng/mL) for 24 h, used as pro-inflammatory stimuli. Vehicle-treated cells were used as control. In competition experiments, the CB2R antagonist (SR144528, 1 μM; Cayman Chemical Company, Ann Arbor, MA, USA) or the CB2R positive allosteric modulator (**EC21a**, 10 μM) was administered 15 min before agonist administration.

### 3.4. MTT (Cell Viability Assay)

Cell viability was evaluated by 3-(4,5-dimethylthiazol-2-yl)-2,5-diphenyltetrazolium bromide (MTT) reagent. Briefly, HMC3 cells were exposed to increasing concentrations of the compound, ranging from 0.1 to 25 µM. After 24 h, 0.5 mg/mL MTT reagent was added to each well, and the cells were incubated for 3 h at 37 °C. Then, 25 µL of the medium was removed from the wells, and 50 µL of DMSO was added. After 10 min incubation at 37 °C, absorbance at OD540 nm was determined with an automated microplate reader (BIO-TEK, Winooski, VT, USA). The percentage of cell viability was calculated as a percentage of vehicle-treated cells used as control.

### 3.5. Statistical Analysis 

HitHunter^®^ cAMP data are shown as % of maximal CP55,940 response (i.e., 100%).Concentration–response curves (CRC) were fit using non-linear regression (3 parameters) and used to calculate EC_50_ or E_max_ (GraphPad, Prism, v. 9.0). Statistical analyses were conducted by one-way analysis of variance (ANOVA), as indicated in the figure legends, using GraphPad. Post-hoc analyses were performed using Tukey’s (one-way ANOVA) test. The homogeneity of variance was confirmed using Bartlett’s test. All results are reported as the mean ± the standard error of the mean (SEM) or 95% confidence interval (CI), as indicated. *p* values < 0.05 were considered significant.

### 3.6. Plasma Stability

Stock solutions of 1 mg/mL **JR22a** and procainamide (used as internal standard, IS) were prepared in CH_3_OH and kept in ice during sample preparation and stored at −20 °C if not in use. The stability was tested in rat and human plasma obtained from Sigma-Aldrich (Milan, Italy). 

**JR22a** (20 µM) was incubated with plasma at 37 °C under gentle shaking up to X h in triplicates. An aliquot of 20 µL was withdrawn from the mixture and diluted to a final volume of 200 µL of a 2 % TCA solution containing procainamide 1 µM at each time point: 10, 30, 60, 120, 240, and 360 min. Samples were kept in ice for 20 min and then centrifugated at 14,000 rpm for 10 min at 4 °C. The supernatant was diluted 1:1 with water and transferred in vials for the analysis. Separation was performed on a reversed-phase Agilent Zorbax SB-C18 column (150 × 2.1 mm, i.d. 3.5 µm, CPS analitica, Milan, Italy), protected by an Agilent Zorbax guard column, kept at 45 °C, by an UltiMate 3000 system (Dionex, Softron GmbH, Germering, Germany) equipped with an autosampler kept at 4 °C working at a constant flow rate (300 µL/min). Each sample (20 µL) was injected into the column, and components were eluted with a 15 min gradient of phase A H_2_O–0.1 % HCOOH (% *v*/*v*) and phase B CH_3_CN–0.1 % HCOOH (% *v*/*v*): 0–6.7 min, from 10% B to 95% B; 6.7–9.7 min isocratic of 95% B; 9.7–9.71 min, from 95% B to 10% B, and then 9.71–15 min of isocratic 10% B. The MS analyses were performed on an LTQ-Orbitrap XL mass spectrometer using an ESI source (Thermo Fisher Scientific, San Jose, CA, USA). Mass spectra were acquired in positive ion mode. The source parameters used are: spray voltage +4.5 kV, capillary temperature 350 °C, capillary voltage +47 V, tube lens offset +120 V, sheath gas 40 a.u., auxiliary gas 5 a.u. The instrument was set up to work in Full MS scan mode in a scan range of m/z 120–900, using a resolution of 30,000 FWHM at *m/z* 400. For the semiquantitative analysis, the extracted ion chromatograms (XICs) relative to the JR22 ion (m/z 789.34511) and the IS (m/z 236.17572) were extracted with an accuracy of 5 ppm, and the AUC was integrated by using the Genesis algorithm of the Qual Browser tool of Xcalibur 4.0. The ratio between the AUC of **JR22a** and the one of the IS was calculated for each point, and the relative percentage with respect to t = 0 min was defined as [(AUC_jr22 tx_/AUC_IStx_)/(AUC_jr22t0_/AUC_ISt0_)] × 100.

### 3.7. Computational Metabolism Prediction

Prediction of **JR22a** metabolism was made through MetaSite 6.0 [16] using the P450 liver model and all CYP models implemented in the software (CYP1A1, CYP1A2, CYP2B6, CYP2C19, CYP2C9, CYP2D6, CYP2E1, CYP3A4, CYP3A5) with reactivity correction, generating 50 conformers at the beginning. Metabolite identification was performed using all the common cytochrome P450 biotransformations as the program default and including all metabolites with MW > 50 Da. 

### 3.8. Docking

All ligands are built and optimized using Maestro, considering for **JR** compounds both cis- conformers of the 4-methyl cyclohexyl group; they were subjected to a Conformational Search (CS) of 1000 steps in a water environment using the Macromodel program. The Monte Carlo algorithm was used with the MMFFs forcefield. The ligands were then minimized using the Conjugated Gradient method to a convergence value of 0.05 kcal/Å∙mol using the same forcefield and parameters as for the CS. The central scaffold of compounds JR22 and JR64, the N-(4-methylcyclohexyl)-2-oxo-1,2-dihydro-1,8-naphthyridine-3-carboxamide was built, and both cis-conformers (bearing the amide moiety in an axial or equatorial position) were subjected to a B3LYP/6-31G** optimization, in order to evaluate the best conformer. Crystallographic structures 6PT0 [17], relative to the active conformation of CB2R, already refined through Maestro, had been used for docking **LV62, EC21a,** CP55,940, and all **JR** compounds using the GOLD program, applying the same procedure described in our previous study on 2-oxo-pyridine derivatives. For docking **LV62**, the region of interest was defined in such a manner that the protein contained all the residues within 10 Å of WIN 55,212-2. All **JR** compounds were subjected to docking in the empty orthosteric cavity, with a scaffold constraint of strength 5 on the **LV62** core of the docked pose; in this way, the orthosteric pharmacophoric portion of **JR** compounds can fit the usual CB2R agonist cavity, whereas the **EC21a**-derived allosteric tail can be free to reach a favorable binding site. In the same condition, also the free calculation, without any scaffold constraint, was performed. The “allow early termination” command was always deactivated. All ligands were submitted to 40 Genetic Algorithm runs using Chemscore, ASP, PLP, and Goldscore fitness functions, clustering the output orientations on the basis of an RMSD distance of 1.5 Å. The default GOLD parameters were used for all other variables. Docking results were analyzed by using Chimera 1.16. 

### 3.9. Molecular Dynamics 

The initial simulation system was built using PDB ID 6PT0 [17], removing cholesterol and palmitic acid and orienting it along the Z axis; chain A was maintained, in addition to chain R, for preserving the interaction of transmembrane region with subunit alpha-1 of guanine nucleotide-binding protein G(i), the only one subunit directly bound to the receptor. The structure was embedded in a bilayer of 500 units of palmitoyl-oleoylphosphatidylcholine (POPC), 251 at the top leaflet and 249 at the bottom leaflet. 27,061 TIP3P water molecules and 0.15 M KCl (including neutralization ions, 126 K+, and 140 Cl) were added to the system, extending 40 Å at the top and bottom of the membrane, and the initial system size was 135.1 × 135.1 × 124.2 Å^3^.

The system was rebuilt in Amber20 using tLeap to generate the topology file, protonation, angles, and dihedrals of the complex, inserting in CB2R the ligand in its docked pose. N- and C-termini of the protein model systems were capped by acetyl and methylamino groups. General Amber force field (GAFF) parameters were assigned to the ligand, while partial charges were calculated using the AM1-BCC method as implemented in the Antechamber suite of AMBER 20. The default particle mesh Ewald method (PME) was used to calculate long-range electrostatic interactions with a coefficient of 0.275 Ǻ. Van der Waals and short-range electrostatic interactions were smoothly truncated at 10.0 Ǻ. The Langevin thermostat and the anisotropic Berendsen barostat were employed to equilibrate the temperature and control the pressure, respectively. Periodic boundary conditions were applied. The time step of the simulations was 2.0 fs with a cutoff of 10 Å for the non-bonded interaction, and SHAKE was employed to keep all bonds involving hydrogen atoms rigid. The system was minimized by ten thousand steps of steepest descent, and conjugate gradient minimization, with harmonic restraints of 50 kcal/mol Ǻ-2 applied on all solute atoms, then by 10,000 steps of minimization without restraints. The heating simulation was run in two phases: at first, a 400 ps simulation kept the system at 100 K in the NVT (constant number of particles, volume, and temperature) ensemble with a force constant of 50 kcal/mol Ǻ-2 on all atoms except solvent, then progressively relaxing lipids (after 160 ps) and ions (after 280 ps); in a second step, the temperature was raised during a further 400 ps MD simulation to 300 K in the NPT ensemble with the same restraining scheme of the first heating. The temperature of 300 K was used in equilibration MD to ensure that the membrane state was well above the melting point of POPC. An equilibration of 10 ns was performed in four stages: in the first of 400 ps, all the protein complexes were restrained with a force constant of 50 kcal mol Ǻ-2 on all complex atoms. In the next 600 ps, the ligand and side chains were relaxed, and for a further 3 ns, only the alpha-carbons were constrained. The last step of 6 ns was an NPT simulation without restraints. The MD trajectories were analyzed using the MD Movie tool of Chimera and the cpptraj module of AMBER 20.

## 4. Conclusions

GPCRs represent one of the most important pharmaceutical drug-target classes because of their regulation of a wide variety of human physiological processes. The rational design of dualsteric ligands is a promising new strategy to obtain fine-tuned GPCR modulation. The availability of a single molecular entity able to simultaneously interact with both the orthosteric and the allosteric receptor sites offer several advantages over monovalent targeting strategies, including a bias in signaling pathway activation, reduced off-target activity, and therapeutic resistance.

Recently we reported the design of two novel series of potential CB2R dualsteric ligands, obtained by connecting through various spacers the pharmacophoric portion of the CB2R PAM **EC21a** to that of the selective CB2R orthosteric agonist **LV62**. In the present work, functional and computational studies allowed us to identify the **JR22a** compound as an effective CB2R dualsteric ligand. Computational studies showed that **JR22a** appears to preserve the orthosteric binding mode and engages in interactions with an unconserved region of the CB2R surface, which was suggested as a potential allosteric binding site [10]. The results also highlighted that the choice of the linker between the two pharmacophoric portions plays a crucial role in the design of bitopic compounds.

**JR22a** was also investigated to evaluate its ability to prevent the inflammatory response in LPS/TNFα stimulated HMC3 cells. The results showed that **JR22a** used at 1 and 10 μM produced significant CB2R-mediated anti-inflammatory effects. Moreover, the co-administration with CB2R PAM **EC21a** did not interfere with the anti-inflammatory effects of **JR22a**, supporting its bitopic behavior. Finally, **JR22a** was found to be stable both in rat and human plasma, and computational metabolism prediction analysis displayed the hepatic stability of the bitopic structure with respect to the metabolic fragmentation.

In conclusion, dualsteric/bitopic ligand **JR22a** can be a valuable tool for a better understanding of the physiological effects related to the bitopic stimulation of CB2R. In particular, **JR22a**, engaging the orthosteric and allosteric sites simultaneously, could produce neuroprotective effects and have beneficial therapeutic applications in diseases when the endogenous tone is reduced.

## Figures and Tables

**Table 1:** Inhibition of Forskolin-Stimulated cAMP.

Compound(s)	pEC_50_ ± SEM (nM)	E_max_ ± SEM (%)	*N*
**CP55,940**	7.5 ± 0.2 (31)	100 ± 6	27
**EC21a**	7.5 ± 0.9 (30)	−14 ± 7 ***	8
**JR14a**	7.6 ± 0.9 (24)	29 ± 5 *** ^^	6
**EC_50_ JR14a + EC21a**	7.0 ± 1 (90)	68 ± 10 ^^^	5
**100 nM SR144528 + JR14a**	7.7 ± 0.9 (19)	30 ± 10 ***	5
**JR64a**	8.6 ± 2 (2.3)	19 ± 6 ***	5
**EC_50_ JR64a + EC21a**	7.3 ± 1 (47)	74 ± 10 ** ^^^ ^††^	5
**100 nM SR144528 + JR64a**	8.5 ± 0.4 (2.9)	43 ± 9	3
**JR22a**	6.1 ± 0.2 (698)	53 ± 6 *** ^^^	14
**EC_50_ JR22a + EC21a**	6.0 ± 0.4 (960)	44 ± 9 *** ^^^	9
**100 nM SR144528 + JR22a**	<5 (>10,000)	49 ± 20 ***	9

CB2R activity was quantified for cAMP inhibition using the DiscoveRx HitHunter assay (CHO hCB2R) in cells treated with compounds for 90 min. Data were fit to a three-parameter non-linear regression model in GraphPad (v. 9). Data are mean ± SEM. *n* number of independent experiments performed in triplicate indicated in the table. ** *p* < 0.01, *** *p* < 0.001 compared to CP55,940; ^^ *p* < 0.01, ^^^ *p* < 0.001 compared to **EC21a**; ^††^ *p* < 0.01 compared to **JR64a,** as determined by two-way ANOVA followed by Tukey’s *post-hoc* test. Data from this Table are graphed in Figure 3 and Figure 4.

## Data Availability

Not applicable.

## Supplementary Materials

The following supporting information can be downloaded at: https://www.mdpi.com/article/10.3390/ijms24032135/s1.
