# Supplementary material for: New Insights into Bitopic Orthosteric/Allosteric Ligands of Cannabinoid Receptor Type 2
Source: Int J Mol Sci. 2023 Jan 21;24(3):2135. doi: 10.3390/ijms24032135 (PMC9917213; doi:10.3390/ijms24032135)
Supplement: Supplementary file 1 [file ijms-24-02135-s001.zip › ijms-2100429-supplementary.pdf]

Supplementary Material

NEW INSIGHTS INTO BITOPIC ORTHOSTERIC/ALLOSTERIC LIGANDS OF CANNABINOID RECEPTOR TYPE 2

Rebecca Ferrisi [1], Beatrice Polini [2], Caterina Ricardi [2], Francesca Gado [3], Kawthar A. Mohamed [4], Giovanna Baron [3], Salvatore Faiella [1], Giulio Poli [1], Simona Rapposelli [1], Giuseppe Saccomanni [1], Giancarlo Aldini [2], Grazia Chiellini [2], Robert B. Laprairie [4,5], Clementina Manera [1,*], Gabriella Ortore [1]

$[^3H]$CP55,940 Binding Assays. We assessed ligand affinity for **JR-22a** at *h*CB$_1$R and *h*CB$_2$R using a $[^3H]$CP55,940 radioligand displacement assay using membranes derived from CHO cells stably expressing either receptor. At *h*CB$_1$R, **JR-22a** was not able to displace $[^3H]$CP55,940 (Figure S1a). At *h*CB$_2$R, **JR-22a** displaced $[^3H]$CP55,940 bound to the receptor (Figure S1b), with a IC$_{50}$ value of 95 nM.

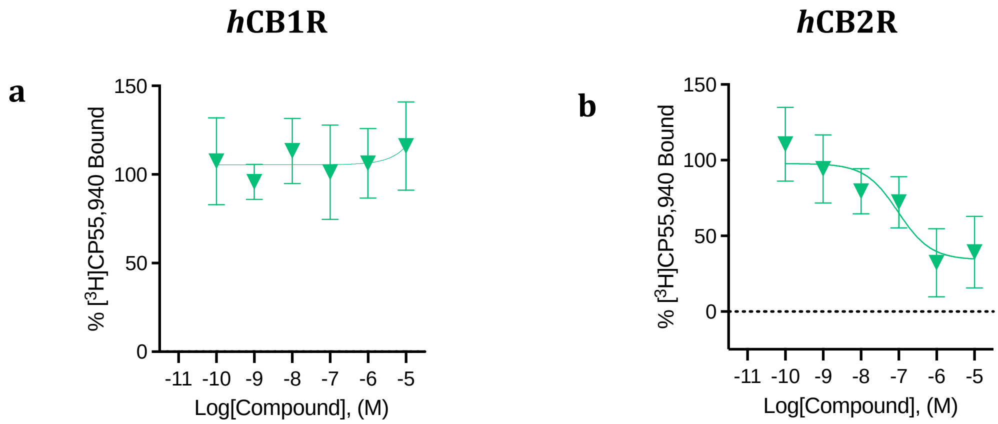

Figure S1. $[^3H]$CP55,940 binding to *h*CB1R and *h*CB2R. Membranes from CHO-K1 cells stably-expressing *h*CB1R **(a)** or *h*CB2R **(b)** were treated with 1 nM $[^3H]$CP55,940 and 0.10 nM-10 µM compounds for 2 h. Data are expressed as %$[^3H]$CP55,940 bound. Data were fitted to a non-linear regression (three-parameter model, GraphPad v. 9.0). Data are mean ± S.E.M. of 3 independent experiments performed in triplicate.

Radioligand Displacement Assay. $[^3H]$CP55,940 (174.6 Ci/mmol) was obtained from PerkinElmer (Guelph, ON). Cells were scraped from flasks, centrifuged, and frozen at −80 °C until required.

This method was conducted as described previously [19,20]. Cells were thawed, diluted in Tris buffer (50 mM Tris−HCl and 50 mM Tris−base), and homogenized in a 1 mL handheld homogenizer. *h*CB1R and *h*CB2R CHO−K1 cell membranes were collected by cavitation in a pressure cell and sedimented by ultracentrifugation. Pellets were resuspended in TME buffer (50 mM Tris−HCl, 5 mM MgCl2, 1 mM EDTA, pH 7.4), and protein concentration was measured via the Bradford method (Bio-Rad Laboratories, Mississauga, ON). Competition binding experiments were conducted with 1 nM [3H]CP55, 940 and Tris binding buffer (50 mM Tris−HCl, 50 mM Tris−base, 0.1% BSA, pH 7.4, 2 mL). Radioligand binding began with the addition of CHO−K1 cell membranes (50 µg protein per sample). Assays were performed for 120 min at 37 °C and stopped by the addition of ice-cold Tris binding buffer, followed by vacuum filtration using a 24-well sampling manifold (Brandel Cell Harvester; Brandel Inc, Gaithersburg, MD, USA). Brandel GF/B filter paper was soaked with wash buffer at 4 °C for at least 24 h. Each filter paper was washed 6 times with 1.2 mL aliquots of Tris-binding buffer then air-dried overnight and submerged in 5 mL of scintillation fluid (Ultima Gold XR, PerkinElmer). Liquid scintillation spectrometry was used to quantify radioactivity. For competition binding experiments, specific binding was equal to the difference in radioactivity with or without 1 µM unlabelled CP55, 940.

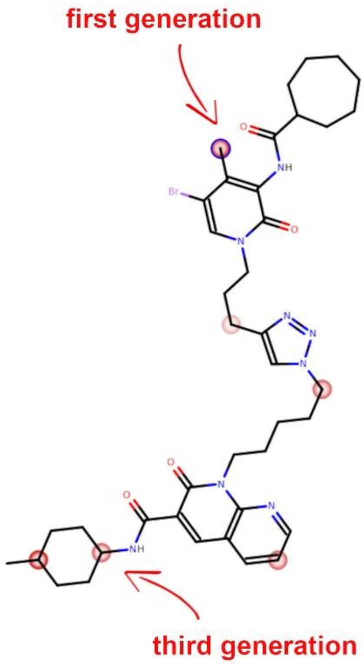

Figure S2. Sites of Metabolism predicted by MetaSite for **JR22a**. Positions involved in main transformations are highlighted. The first generation of metabolites derive by hydroxylation, oxydation or dehydrogenation of methyl substituent of the oxopyridine moiety in position 4. The first cleavage of the molecule was detected during the third generation of metabolites, at the carbon adjacent to N of the 4-methylcyclohexylcarboxamide moiety.

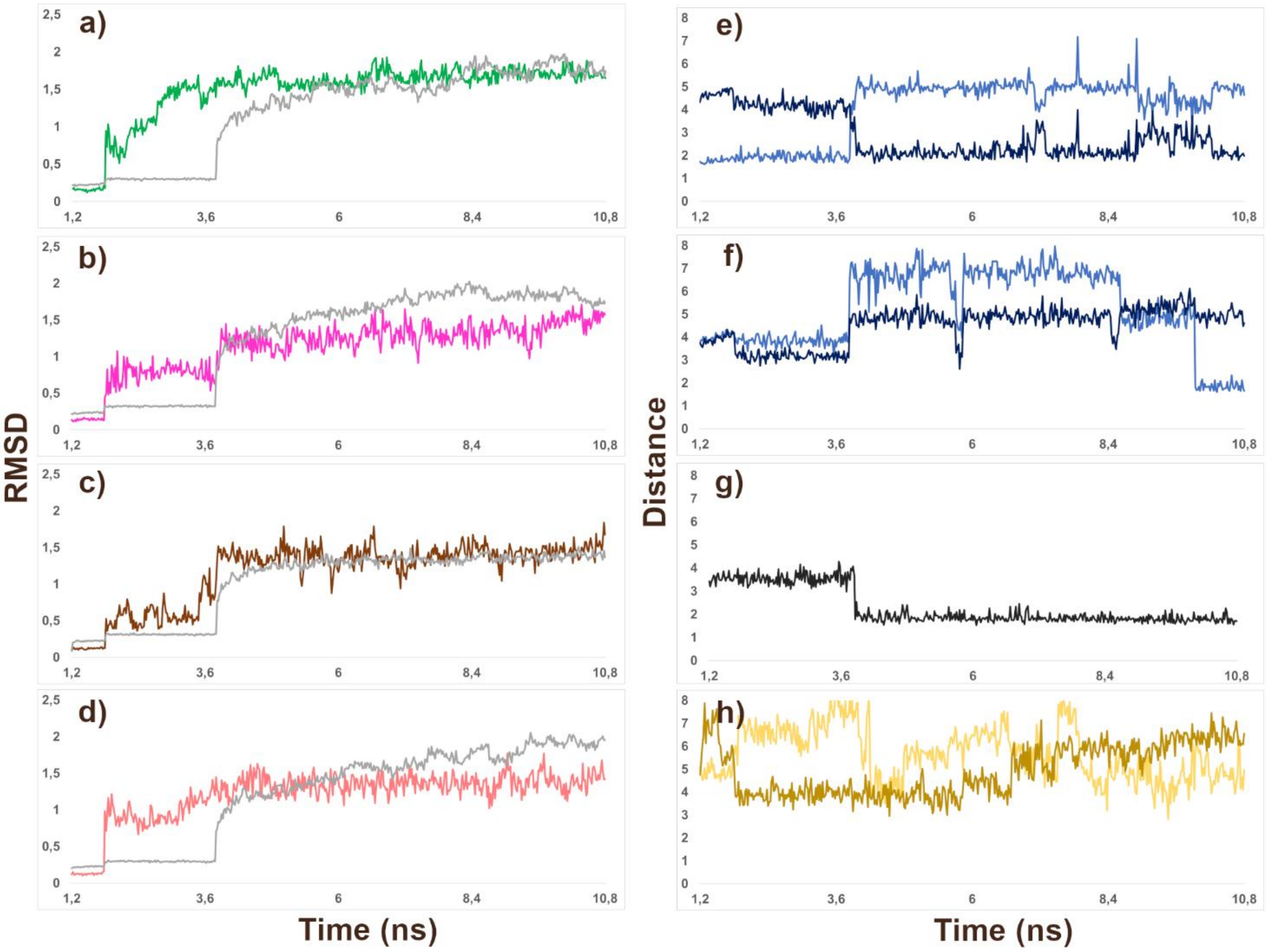

Figure S3. root-mean-square deviation (RMSD) of α-carbons and ligands heavy atoms. on the left, RMSD (Å) of alpha-carbons (grey) and heavy atoms of: a) **FD22a** (green), b) **JR22a** (magenta), c) **JR64a** (brown), d) **JR14a** (coral); heating time is not reported. On the right, distances (Å) as a function of time (ns) between: e) Ser193 and amidic NH (dark blue) or C=O (light blue) of **FD22a**; f) Ser193 and amidic NH (dark blue) or C=O (light blue) of **JR22a**; g) Tyr190 and oxopyridine-O of **JR64a**; h) Gln32 - triazole-N (brown) and Gln276 - oxopyridine-O (gold).

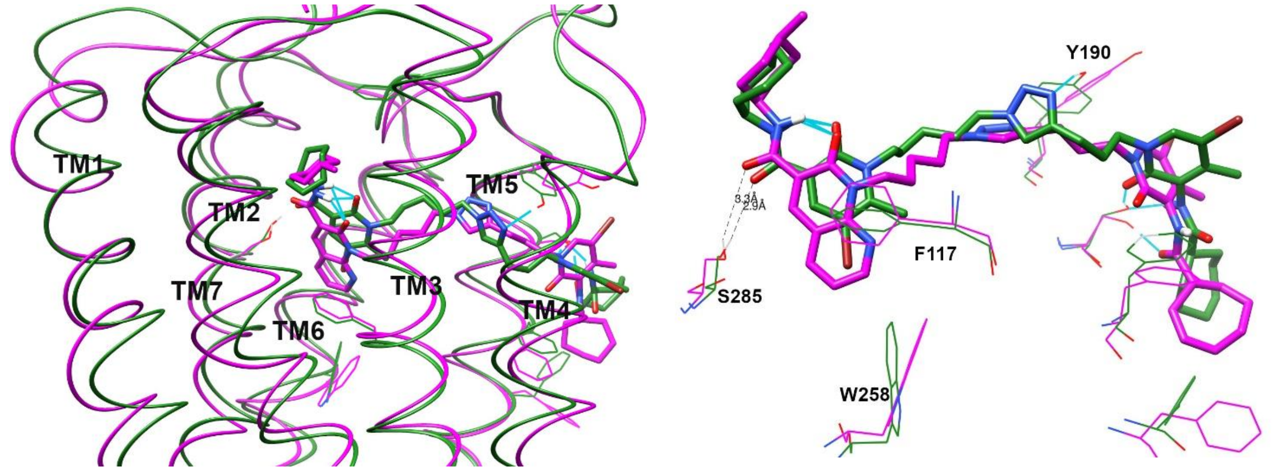

Figure S4. Superposition of **FD22a** (green) and **JR22a** (magenta) poses in CB2R: left, receptor front view; right, details of interactions with orthosteric site.
